# Fatty acid binding profile of the liver X receptor α[S]

**DOI:** 10.1194/jlr.M072447

**Published:** 2017-01-31

**Authors:** Shimpi Bedi, Genesis Victoria Hines, Valery V. Lozada-Fernandez, Camila de Jesus Piva, Alagammai Kaliappan, S. Dean Rider, Heather A. Hostetler

**Affiliations:** Department of Biochemistry and Molecular Biology, Boonshoft School of Medicine, Wright State University, Dayton, OH 45435

**Keywords:** human liver X receptor α, peroxisome proliferator-activated receptor, transcription factor, endogenous ligand, medium-chain fatty acid, long-chain fatty acid, long-chain fatty acyl-coenzyme A

## Abstract

Liver X receptor (LXR)α is a nuclear receptor that responds to oxysterols and cholesterol overload by stimulating cholesterol efflux, transport, conversion to bile acids, and excretion. LXRα binds to and is regulated by synthetic (T-0901317, GW3695) and endogenous (oxysterols) ligands. LXRα activity is also modulated by FAs, but the ligand binding specificity of FA and acyl-CoA derivatives for LXRα remains unknown. We investigated whether LXRα binds FA or FA acyl-CoA with affinities that mimic in vivo concentrations, examined the effect of FA chain length and the degree of unsaturation on binding, and investigated whether FAs regulate LXRα activation. Saturated medium-chain FA (MCFA) displayed binding affinities in the low nanomolar concentration range, while long-chain fatty acyl-CoA did not bind or bound weakly to LXRα. Circular dichroic spectra and computational docking experiments confirmed that MCFA bound to the LXRα ligand binding pocket similar to the known synthetic agonist of LXRα (T0901317), but with limited change to the conformation of the receptor. Transactivation assays showed that MCFA activated LXRα, whereas long-chain FA caused no effect. Our results suggest that LXRα functions as a receptor for saturated FA or acyl-CoA of C_10_ and C_12_ in length.

Nuclear hormone receptors are ligand-activated transcription factors that mediate the transcriptional effects of steroid, thyroid, and retinoid hormones (1–4). Among the dietary nutrients that act as ligands and serve as signaling molecules to regulate cellular metabolism are oxysterols and FAs (5–7). These compounds directly bind to the nuclear receptor ligand-binding domain (LBD) and induce conformational changes to trigger the exchange of corepressors with the coactivators leading to the repression or activation of the target genes (8, 9). Liver X receptors (LXRs) are ligand-activated nuclear receptors belonging to the steroid hormone receptor superfamily that specifically bind to and are activated by oxysterols. Both isoforms of LXR form heterodimers with the retinoid X receptor (RXR), which then bind to specific DNA elements to regulate gene transcription. The LXR-RXR complex exhibits basal levels of transcription in the absence of a ligand. Upon ligand activation, LXRs act as transcription factors to regulate the expression of genes involved in cholesterol transport, lipid metabolism, and carbohydrate metabolism. There are two LXR isoforms: the α isoform is found in metabolically active tissue, such as liver and kidney, whereas the β isoform is ubiquitously expressed (10). Although both isoforms are involved in regulating cholesterol homeostasis, the α isoform is the predominant isoform that functions as a master hepatic lipogenic transcription factor (11).

In LXRα knockout mice, the CYP7a1 gene (which is involved in cholesterol metabolism) is downregulated, resulting in accumulation of cholesterol in the liver. Genes involved in hepatic FA biosynthesis, such as SREBP-1, stearoyl-CoA desaturase, and FAS, are also downregulated in LXRα-deficient mice, and LXRβ was unable to compensate for this loss of LXRα. In LXRβ-deficient mice, expression of the above genes remains unaffected (12, 13). Furthermore, patients with nonalcoholic fatty liver disease and hepatitis C virus-induced steatosis have elevated levels of LXRα and its target gene involved in lipogenesis (14–16). Not surprisingly, LXRs are attractive drug targets for the treatment of diabetes and metabolic disorders (17–19).

Although oxysterols are classical endogenous ligands of LXRs, FAs have been reported to inhibit oxysterol binding to LXR. The inhibition depends on the degree of unsaturation of the FAs; polyunsaturated FAs are more potent inhibitors of oxysterol binding compared with monounsaturated FAs, suggesting that FAs or fatty acyl-CoAs may directly bind LXRα (20–23). Furthermore, LXRα can form a heterodimeric pair with PPARα (24), and each of the two proteins individually responds to FAs (25, 26). This creates complexity in understanding and characterization of individual signaling pathways. To differentiate the direct and indirect effects of PPAR ligands (FAs) on LXRα, it is important to quantify the binding affinities of FA binding to LXRα. The main goal of this study was to test the hypothesis that LXRα serves as a FA receptor through investigating the kinetics of FA binding to LXRα.

## MATERIALS AND METHODS

### Purification of recombinant human LXRα

Plasmids for full-length human (h)LXRα recombinant protein expression were transformed into Rosetta 2 competent cells. Protein was purified through affinity chromatography with the GST tag and on column digestion as described. Protein concentrations were estimated by the Bradford assay (Bio-Rad, Hercules, CA). Protein purity was determined by SDS-PAGE followed by Coomassie blue staining and Western blotting (27).

### Reagents

Fluorescent FAs (BODIPY-C16 and BODIPY-C12) were purchased from Molecular Probes, Inc. (Eugene, OR). BODIPY C12-CoA and BODIPY C16-CoA were synthesized and purified by HPLC, as previously described, and found to be >99% unhydrolyzed (28). All other putative ligands were from Sigma-Aldrich (St. Louis, MO).

### Fluorescent ligand binding assays

Fluorescent ligand (BODIPY C16, BODIPY C12, BODIPY C12-CoA, or BODIPY C16-CoA) binding measurements were performed using 0.1 μM LXRα with increasing concentrations of fluorescent ligand in PBS, pH 7.4. Fluorescence emission spectra (excitation, 465 nm; emission, 490–550 nm) were obtained at 24°C with a PC1 photon counting spectrofluorometer (ISS Inc., Champaign, IL), corrected for background (protein only and fluorescent ligand only), and maximal intensities used to calculate the apparent dissociation constant (*K_d_*) (28, 29). All ligand concentrations were below the critical micelle concentrations and were delivered using ethanol as a solvent.

### Displacement of bound fluorescent BODIPY C16-CoA by nonfluorescent ligands

To further examine whether FAs could bind LXRα directly and displace a fluorescent ligand, putative ligands were tested in a displacement assay using recombinant LXRα and BODIPY-labeled C16-CoA in PBS, pH 7.4. LXRα (0.1 μM) was mixed with 0.1 μM of BODIPY C16-CoA and the maximal fluorescence intensity was measured. The effect of increasing concentrations of FAs or fatty acyl-CoA was measured as a quenching in fluorescence of BODIPY C16-CoA. Emission spectra were obtained at 24°C and corrected for background as described above for BODIPY. Changes in fluorescence intensity were used to calculate the inhibition constant (*K_i_*) values (28, 29).

### Quenching of LXRα aromatic amino acid residues by nonfluorescent ligands

The direct binding of LXRα to nonfluorescent ligands was determined by quenching of intrinsic LXRα aromatic amino acid fluorescence. Briefly, LXRα (0.1 μM) was titrated with increasing concentrations of ligand in PBS, pH 7.4. Emission spectra from 300 to 400 nm were obtained at 24°C upon excitation at 280 nm with a PC1 photon-counting spectrofluorometer (ISS Inc.). Data were corrected for background and inner filter effects, and maximal intensities were used to calculate the apparent dissociation constant (*K_d_*) (28, 29).

### Secondary structure determination: effect of ligand binding on LXRα circular dichroism

Circular dichroic spectra of hLXRα [0.6 μM in 600 μM HEPES (pH 8.0), 24 μM dithiothreitol, 6 μM EDTA, 6 mM KCl, and 0.6% glycerol] were taken in the presence and absence of FAs and fatty acyl-CoA (0.6 μM) with a J-815 spectropolarimeter (Jasco Inc., Easton, MD). Ligand stock solutions were prepared in ethanol or KH_2_PO_4_ as vehicle. Spectra were recorded from 260 to 187 nm with a bandwidth of 2.0 nm, sensitivity of 10 millidegrees, scan rate of 50 nm/min, and a time constant of 1 s. Ten scans were averaged and percent compositions of α-helices, β-strands, turns, and unordered structures were estimated using the CONTIN/LL program of the software package CDPro (27–31).

### Mammalian expression plasmids

Human (h)PPARα and hLXRα from polyhistidine tag (6xHis)-GST-hPPARα and 6xHis-GST-hLXRα were transferred into the multiple cloning site of pSG5 (Stratagene; *Bam*H1-end-filled *Bgl*II) to produce pSG5-hPPARα and pSG5-hLXRα, respectively, as described (27). The human (h)SREBP-1c minimal promoter (−520 to −310) containing the LXR response element (LXRE) (32) was cloned into the pGEM-T easy vector (Promega, Madison, WI) and subsequently transferred into *Kpn*I-*Xho*I sites of pGL4.17 (Promega) to produce hSREBP-1c-pGL4.17. All plasmid constructs were confirmed by DNA sequencing.

### Cell culture and transactivation assay

COS-7 cells (ATCC, Manassas, VA) were grown in DMEM supplemented with 10% fetal bovine serum (Invitrogen, Grand Island, NY) at 37°C with 5% CO_2_ in a humidified chamber. Cells were seeded onto 24-well culture plates and transfected with 0.4 μg of each full-length mammalian expression vector (pSG5-hPPARα or pSG5-hLXRα) or empty vector (pSG5), 0.4 μg of the LXRE LUC reporter construct (hSREBP-1c-pGL4.17), and 0.04 μg of the internal transfection control plasmid, pRL-CMV (Promega) with Lipofectamine™ 2000 (Invitrogen). Following transfection incubation, the serum-free DMEM was added for 2 h, ligands (10 μM) were added, and the cells were grown for an additional 20 h. FAs were added as a complex with BSA, as described (27, 28, 31). Firefly luciferase activity, normalized to *Renilla* luciferase (for transfection efficiency), was determined with the dual luciferase reporter assay system (Promega) and measured with a SAFIRE2 microtiter plate reader (Tecan Systems, Inc., San Jose, CA). The sample with no ligand was arbitrarily set to 1.

### Molecular docking

In silico docking of ligands was performed using the LXRα LBD extracted from the LXRα-RXRβ complex [Protein Data Bank (PDB) entry 1UHL]. AutoDock Vina 1.1.2 and FlexiDock module available on SYBYL-X 2.0 (Tripos, St. Louis, MO) were used to determine the binding free energies of receptor-ligand binding as described (31).

### Statistical analysis

Data were analyzed by SigmaPlot™ (Systat Software, San Jose, CA) using the ligand binding macro (one site saturation). One-way ANOVA was used to evaluate overall significance. All results are expressed as mean ± SE. The confidence limit of *P* < 0.05 was considered statistically significant (27, 28, 31).

## RESULTS

### Protein expression and purification

Full-length recombinant hLXRα protein was purified according to the established laboratory protocol, as described (27). The protein with a molecular mass of 51,768 Da migrated at approximately 50 KDa size on a Coomassie blue-stained gel and was determined to be >85% pure. Western blot analyses using antibodies for LXRα confirmed that the 50 KDa band was full-length untagged LXRα (data not shown).

### Binding of fluorescent FA and fatty acyl-CoA to LXRα

**Fig. 1.**
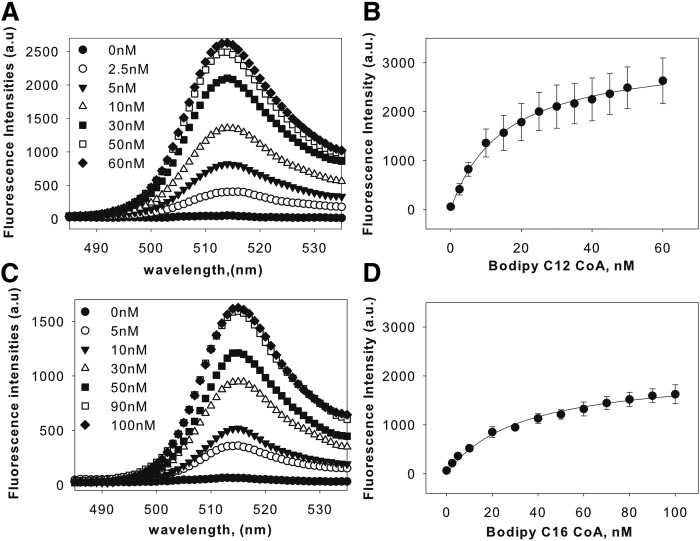
LXRα binds fluorescently labeled saturated fatty acyl-CoA. A: Corrected fluorescence emission spectra of 0.1 μM LXRα titrated with 0 (filled circles), 2.5 (open circles), 5 (filled triangles), 10 (open triangles), 30 (filled squares), 50 (open squares), and 60 nM (filled diamonds) of BODIPY C12-CoA upon excitation at 465 nm, demonstrating that the enhanced fluorescence intensity of BODIPY C12:0-CoA is a result of direct binding with LXRα. B: Plot of LXRα maximal fluorescence emission as a function of BODIPY C12:0-CoA. C: Corrected fluorescence emission spectra of 0.1 μM LXRα titrated with 0 (filled circles), 5 (open circles), 10 (filled triangles), 30 (open triangles), 50 (filled squares), 90 (open squares), and 100 nM (filled diamonds) of BODIPY C16-CoA upon excitation at 465 nm demonstrating that the enhanced fluorescence intensity is a result of binding to LXRα. D: Plot of LXR maximal fluorescence emission as a function of BODIPY C16-CoA.

Because FA and FA acyl-CoA are not inherently fluorescent, BODIPY was conjugated to the ligands for use in the protein-ligand binding studies. BODIPY fluorophores are a safer cost-effective alternative to radioligands that are highly photostable and possess a high fluorescence quantum yield. Because BODIPY compounds have low solubility in aqueous buffers, a low concentration of BODIPY C16:0-CoA (25 nM) was used for binding experiments. In an aqueous buffer without protein, BODIPY fluorescence is quenched and does not yield fluorescence. BODIPY FA binding to the protein enhances the quantum yield of the fluorophore. We used this property to evaluate the binding of fluorescent BODIPY FAs or BODIPY FA acyl-CoA to recombinant LXRα by monitoring the fluorescence emission spectra of the fluorescent ligands in the presence and absence of LXRα. Titration of LXRα with BODIPY C12-CoA or BODIPY C16-CoA resulted in increased fluorescence with an emission maximum near 515 nm, which saturated near 50 nM (**Fig. 1A, B**) and 90 nM (Fig. 1C, D), respectively, suggesting high affinity binding. Based on the quantification of the increase in the quantum yield, the apparent binding constants (*K_d_*) were determined to be 15 ± 6 nM for BODIPY C12-CoA and 32 ± 7 nM for BODIPY C16-CoA. This indicates that BODIPY C12:0-CoA and BODIPY C16:0-CoA can bind LXRα as high affinity ligands. The corresponding FAs showed small changes in the fluorescence intensities, suggesting that these molecules bound relatively more weakly compared with their CoA derivatives (data not shown). The binding of C12:0-CoA, but not C12:0-FA, was further confirmed through using aromatic residues (Tyr/Trp) in LXRα as intrinsic donor and BODIPY-labeled ligands as the corresponding acceptor in a Forster resonance energy transfer assay. Forster resonance energy transfer occurred with the addition of BODIPY C12:0-CoA, but not the corresponding C12:0-FA, to LXRα (supplemental Fig. S1). Taken together, our results show that BODIPY C12:0-CoA and BODIPY C16:0-CoA can bind as high affinity ligands to LXRα.

### Binding of endogenous FA and fatty acyl-CoA to LXRα: displacement of bound BODIPY C16-CoA

**Fig. 2.**
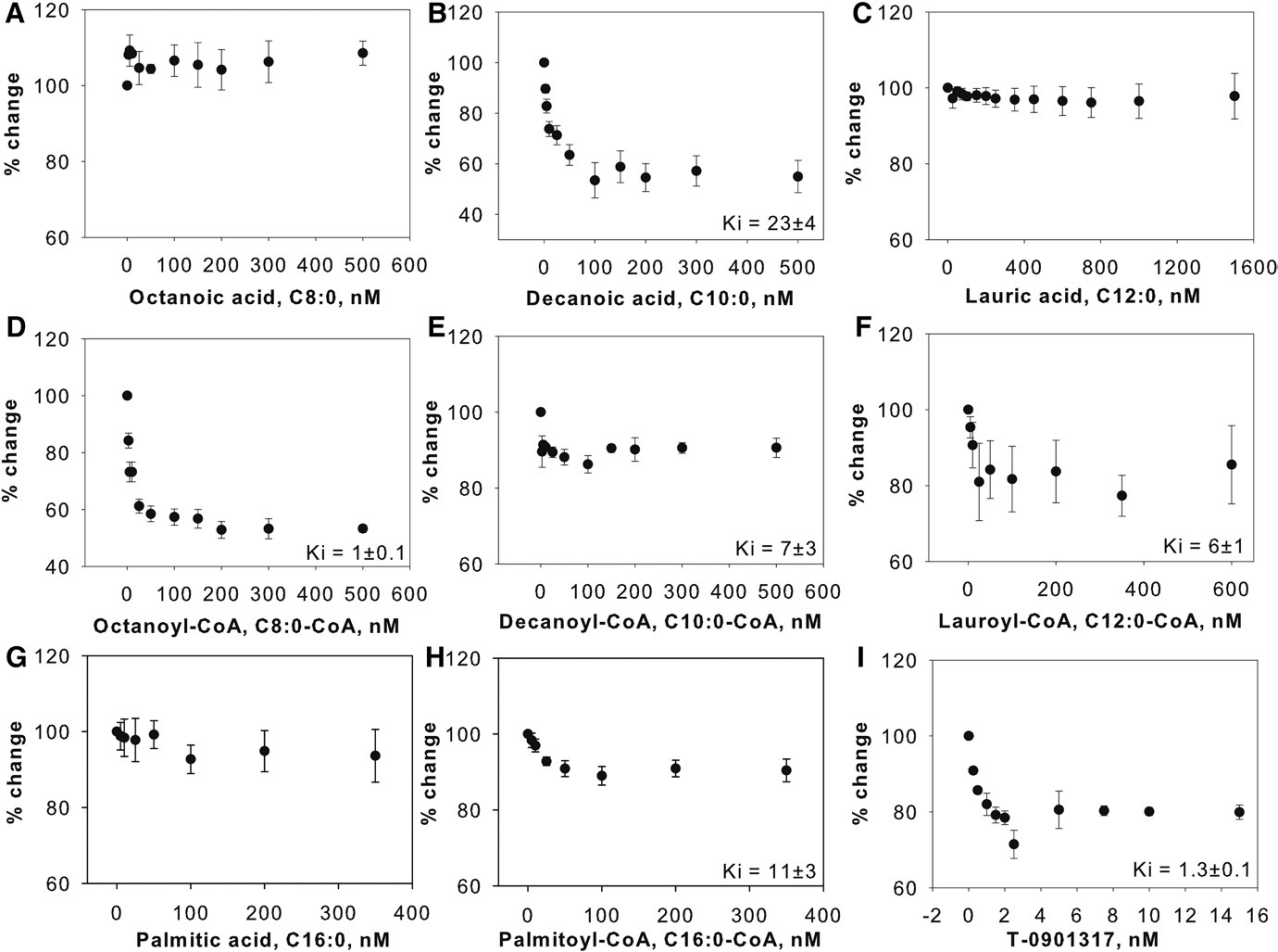
Displacement assay of BODIPY C16:0-CoA bound LXR. BODIPY C16:0-CoA bound to LXRα was displaced with naturally occurring FAs or fatty acyl-CoA. The fall in fluorescence due to displacement of BODIPY C16-CoA from LXRα is expressed as percent changes when titrated with the following ligands: octanoic acid (A), decanoic acid (B), lauric acid (C), octanoyl-CoA (D), decanoyl-CoA (E), lauroyl-CoA (F), palmitic acid (G), palmitoyl-CoA (H), and T-0901317 (I). Data are presented as percent change in fluorescence intensity of BODIPY C16-CoA at 515 nm plotted as a function of ligand concentrations. All values are the average of at least three independent determinations. Error bars represent SE.

To determine the ligand specificity of LXRα for FAs, FA and fatty acyl-CoA of different chain lengths and degree of unsaturation were examined for their ability to displace BODIPY C16:0-CoA from the LXRα ligand binding pocket. The BODIPY C16:0-CoA-LXRα complex was titrated with increasing concentrations of nonfluorescent FA or fatty acyl-CoA until the effect plateaued, and the decrease in fluorescent intensity as the fluorescent lipid was displaced was used to calculate the efficiency (*K_i_*) of the nonfluorescent ligand. By comparing the percent displacement of a variety of FAs or fatty acyl-CoA for a given concentration range, the relative efficiencies of these lipids were distinguished. Whereas decanoic acid, octanoyl-CoA, and lauroyl-CoA caused a 20–50% decrease in the BODIPY fluorescence (**Fig. 2B, D, F**), other ligands had a smaller effect (Fig. 2A, C, E, G, H). Of all FAs and fatty acyl-CoA tested, decanoic acid and octanoyl-CoA showed the highest degree of displacement (Fig. 2B, D). Long-chain FAs were not able to displace BODIPY C16:0-CoA at concentrations as high as 1,600 nM, suggesting that these ligands might bind poorly or not at all to LXRα (supplemental Fig. S2B, C). By comparison, LXR agonists, T-0901317 and 22 (R) hydroxycholesterol (positive controls), displaced the LXRα bound BODIPY C16-CoA by 30% and 50%, respectively (Fig. 2I, supplemental Fig. S2A). These results demonstrate that LXRα preferentially binds medium-chain fatty acyl-CoA, and these ligands compete to some extent for binding to the same site on LXRα as BODIPY fatty acyl-CoA. Comparison among the *K_i_* values suggests that the order of the binding affinities of the studied ligands is: 22 (R) hydroxycholesterol and T-0901317 > octanoyl-CoA > lauroyl-CoA > palmitoyl-CoA > lauric acid and decanoyl-CoA (**Table 1**, supplemental Table S1).

**TABLE 1.** Affinity of hLXRα for nonfluorescent ligands determined by quenching of hLXRα

Ligand	Chain Length:Double Bonds (Position)	*K_d_* (nM)	*K_i_* (nM)
Octanoic acid	C8:0	ND	ND
Octanoyl-CoA	C8:0	15 ± 5	1 ± 0.1
Decanoic acid	C10:0	15 ± 6	23 ± 4
Decanoyl-CoA	C10:0	N.D.	7 ± 3
Lauric acid	C12:0	11 ± 4	19 ± 18
Lauroyl-CoA	C12:0	18 ± 8	6 ± 1
Palmitic acid	C16:0	ND	ND
Palmitoyl-CoA	C16:0	14 ± 7	11 ± 3
T-0901317	—	4 ± 1	1.3 ± 0.1

Aromatic amino acid fluorescence (*K_d_*) and ligand efficiencies determined by displacement of hLXRα-bound BODIPY C16-CoA (*K_i_*). Values represent the mean ± SE (n ≥ 3). ND, not determined.

### Binding of endogenous FA and fatty acyl-CoA to LXRα: quenching of intrinsic aromatic amino acid fluorescence

**Fig. 3.**
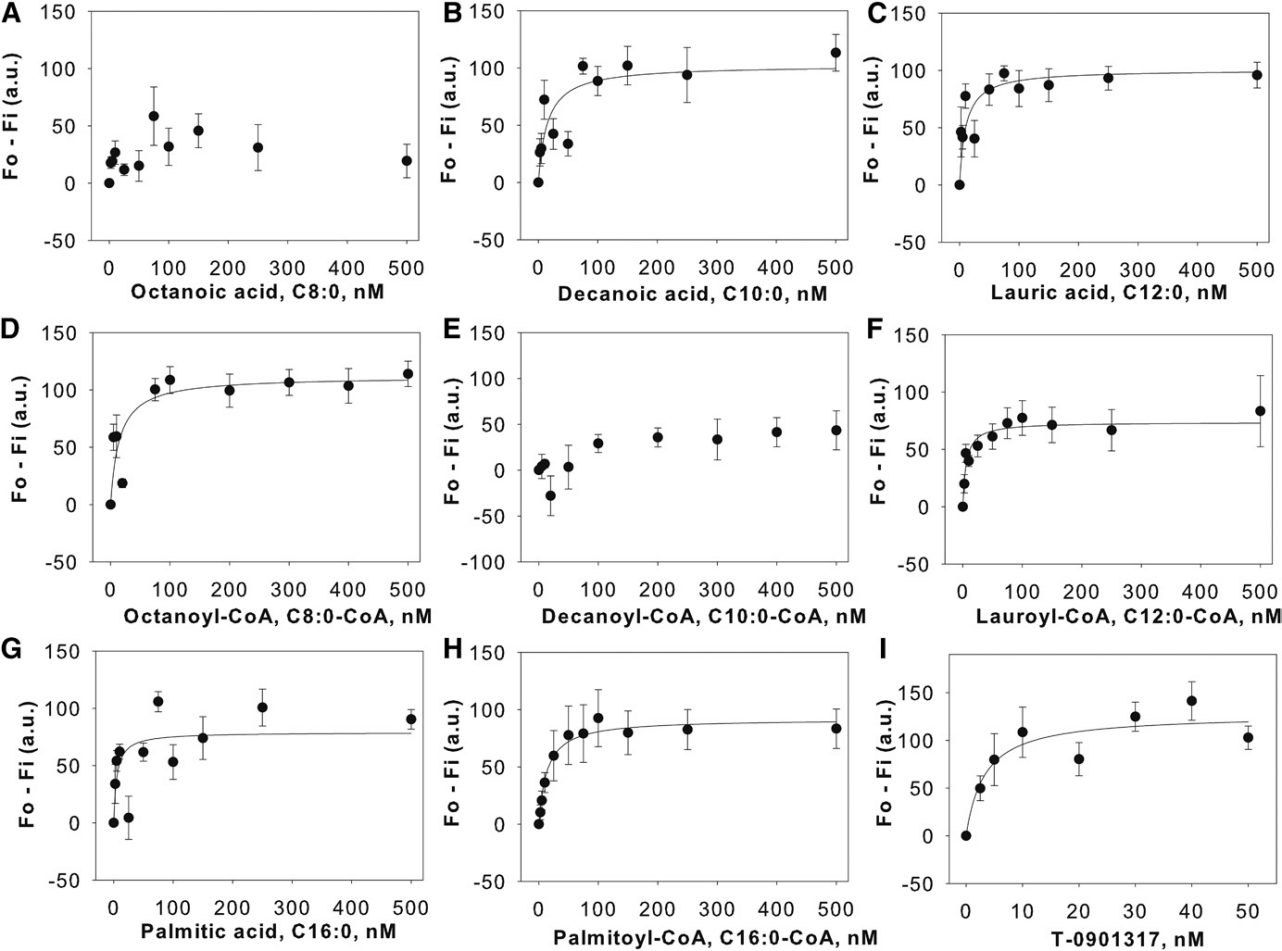
Interaction of naturally-occurring FAs and fatty acyl-CoA with LXRα. Direct binding assay based on quenching of LXRα aromatic amino acid fluorescence emission when titrated with the following ligands: octanoic acid (A), decanoic acid (B), lauric acid (C), octanoyl-CoA (D), decanoyl-CoA (E), lauroyl-CoA (F), palmitic acid (G), palmitoyl-CoA (H), and T-0901317 (I). Data are presented as the change in fluorescence intensity (F_0_-F_i_) plotted as a function of ligand concentration. All values represent mean ± SE, n ≥ 3.

To verify that FAs bind LXRα, we tested these ligands in an intrinsic quenching assay whereby the ability of FA or fatty acyl-CoA to bind LXRα was monitored by fluorescence spectroscopy. Affinities of endogenous FA and fatty acyl-CoA were determined by monitoring the quenching of LXRα aromatic amino acid emission. With excitation at 280 nm, the intrinsic fluorescence of LXRα was observed with a maximum emission at 342 nm. Purified recombinant LXRα (100 nM) was incubated with candidate ligands in a screen of medium-chain saturated FAs, monounsaturated long-chain FAs, polyunsaturated FAs, and the corresponding fatty acyl-CoA derivatives. Titration with octanoic acid (**Fig. 3A**) did not result in decreased LXRα fluorescence. However, addition of decanoic acid and lauric acid resulted in decreased fluorescence with the change in fluorescence intensity plateauing off at approximately 100 nM (Fig. 3B, C). Similar to medium-chain FAs (MCFAs), the apparent *K_d_* values of the remaining ligands binding to LXRα were measured and are listed in Table 1. Titration of LXRα with monounsaturated and polyunsaturated FA yielded no significant quenching of the intrinsic fluorescence, suggesting weak binding or no binding (supplemental Fig. S3). Binding with T-0901317 and 25-hydroxycholesterol (positive controls) yielded binding curves that were sharply saturable with the maximal changes in the intensities at 10 nM and 100 nM, respectively (Fig. 3I, supplemental Fig. S3L). The *K_d_* values of unlabeled C12:0-CoA obtained from the intrinsic quenching is consistent with the value obtained with BODIPY-labeled ligand. However, the *K_d_* values of C16:0-CoA differ between the two assays (Table 1). Because quenching of intrinsic protein fluorescence is a more direct method for the determination of the binding affinity, it provides a more accurate measure of ligand binding. Despite differences between the fluorescent and nonfluorescent methods to measure the apparent *K_d_* values of the ligands, our findings suggest that FAs bind LXRα at nanomolar concentrations. The observed decrease in the intrinsic fluorescence may be a result of direct interaction of LXRα aromatic amino acids with the ligands tested or ligand-induced conformational changes bringing the aromatic amino acids in close proximity to the ligand.

### Effect of endogenous FAs and fatty acyl-CoAs on hLXRα secondary structure

**Fig. 4.**
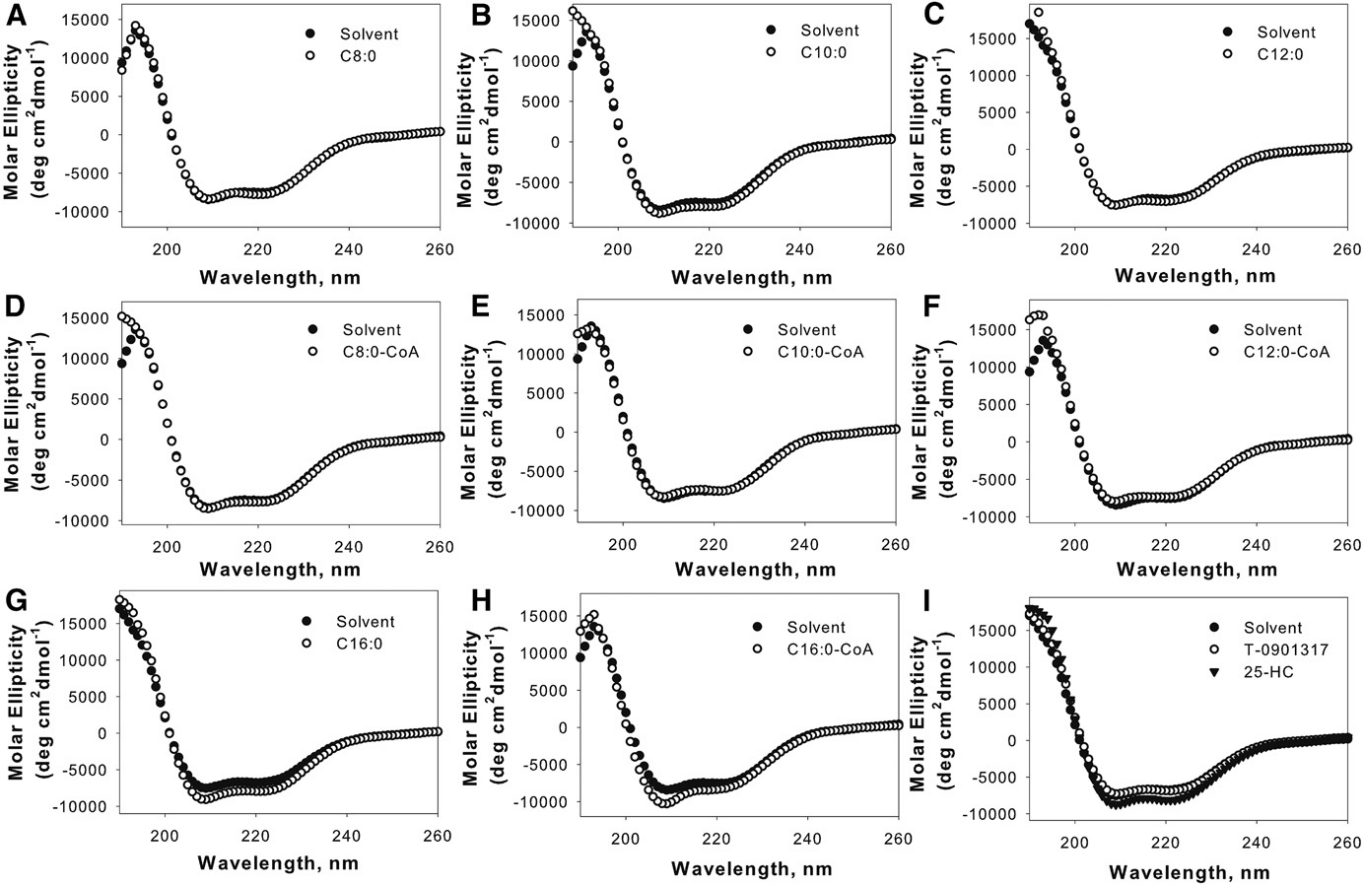
Far UV circular dichroic spectra of LXRα in the absence (filled circles) and presence of added ligand at a concentration of 0.6 μM: octanoic acid, C8:0 (open circles) (A) or octanoyl-CoA, C8:0-CoA (open circles) (D); decanoic acid, C10:0 (open circles) (B) or decanoyl-CoA, C10:0-CoA (open circles) (E); lauric acid, C12:0 (open circles) (C) or lauroyl-CoA, C12:0-CoA (open circles) (F); palmitic acid, C16:0 (open circles) (G) or palmitoyl-CoA, C16:0-CoA (open circles) (H); T-0901317 (open circles) or 25-hydroxycholesterol (25-HC) (filled triangle) (I). Each spectrum represents an average of 10 scans for a given representative spectrum from at least three replicates.

A hallmark of ligand-induced nuclear receptors is the ability of ligand to induce conformational changes in the secondary structure of the proteins. Changes in LXRα intrinsic fluorescence as a result of ligand binding suggested that these changes may correlate with secondary structure changes of the protein. Circular dichroism was used to quantitatively measure changes in the LXRα circular dichroic spectrum due to FA and fatty acyl-CoA binding. **Figure 4** shows the far UV circular dichroic spectrum of LXRα in the absence or presence of the ligands tested. The LXRα spectrum exhibited a large positive peak at 192 nm and two negative peaks at 207 and 222 nm. Quantitative analysis using the CDPro software suggested the presence of 26% α-helical, 22% β-structure, 20% turns, and 32% unordered structures in unliganded-LXRα (**Table 2**). In relation to the ligand-free state, addition of FAs and fatty acyl-CoA caused changes in molar ellipticity at 192, 207, and 222 nm (Fig. 4A–H). The calculated structure (Table 2) showed that C16:0-CoA produced an increase in content and size of the α-helix region. No statistically significant changes were observed with other FAs and fatty acyl-CoA, although small changes in the circular dichroic spectra were evident with C8:0-CoA, C10:0, C12:0-CoA, and C16:0 (Fig. 4B, D–G). Changes observed with these ligands were clearly different from those produced by the solvent. Significant changes in β-sheet content were observed with C8:0-CoA and C10:0, in agreement with the fact that both ligands resulted in changes in intrinsic fluorescence of LXRα. Circular dichroic spectral shifts observed with C12:0 and C16:0 were limited to turns and unordered structures (Table 2). T-0901317, a higher affinity LXR ligand, caused a smaller shift in the circular dichroic spectrum compared with 25-hydroxycholesterol (Fig. 4I). Although we did not detect significant binding of palmitoleic acid and eicosapentaenoic acid to LXRα, small structural changes were observed with these ligands. No significant differences were observed with the polyunsaturated FAs tested (supplemental Table S2).Taken together, these results suggest that FAs and fatty acyl-CoA binding to LXRα causes reorganization of the protein structure with subtle differences observed between various ligands tested.

**TABLE 2.** Secondary structures of hLXRα protein in the presence of FAs and fatty acyl-CoAs

Ligand	α-Helix Regular (%)	α-Helix Distort (%)	β-Sheet Regular (%)	β-Sheet Distort (%)	Turns (%)	Unordered (%)
Ethanol	13.9 ± 0.4	11.9 ± 0.2	13.3 ± 0.3	8.8 ± 0	19.6 ± 0.8	32.4 ± 1.5
C8:0	14.4 ± 0.5	12.0 ± 0.2	12.5 ± 0.6	8.5 ± 0.1	19.6 ± 0.2	32.8 ± 0.5
C8:0-CoA	14.2 ± 1.1	11.5 ± 0.8	15.8 ± 0.5*^c^*	8.9 ± 0.5	19.8 ± 1.3	32.2 ± 3.1
C10:0	13.6 ± 0.6	11.5 ± 0.1	15.3 ± 0.5*^b^*	9.2 ± 0.1*^a^*	21.2 ± 0.1	29.0 ± 0.2
C10:0-CoA	15.3 ± 2.7	11.7 ± 0.4	15.7 ± 1.5*^a^*	8.1 ± 0.8	17.7 ± 3.4	36.7.0 ± 6.4
C12:0	12.1 ± 1.6	9.9 ± 0.3	17.9 ± 0.6	10.5 ± 0.2	20.5 ± 0.1*^a^*	28.9 ± 0.4*^a^*
C12:0-CoA	13.9 ± 0.6	11.3 ± 0.6	14.5 ± 1.2	9.6 ± 0.3	19.9 ± 1	30.6 ± 1.4
C16:0	13.7 ± 1.7	11.4 ± 1.2	15.9 ± 3.9	9.5 ± 0.9	22.1 ± 0.8*^a^*	27 ± 2.5
C16:0-CoA	17.2 ± 2.5	12.8 ± 0.8	12.6 ± 3.7	8.1 ± 0.7	18.1 ± 2.8	35.2 ± 4.8
25-HC	14.7 ± 0.3*^b^*	11.9 ± 0.3*^b^*	12.9 ± 0.9	8.9 ± 0.3	21.4 ± 0.1*^b^*	30.1 ± 0.4*^a^*
T0901317	12.1 ± 1.3	10.5 ± 0.6	16.8 ± 1.5	9.7 ± 0.3	21.3 ± 0.2*^b^*	29.4 ± 0.2*^b^*

Significant difference between hLXRα with solvent compared with the absence or presence of FAs or fatty acyl-CoA (in ethanol) determined by *t*-test. 25-HC, 25-hydroxycholesterol.

a *P* < 0.05.

b *P* < 0.01.

c *P* < 0.001.

### Docking of ligands

**Fig. 5.**
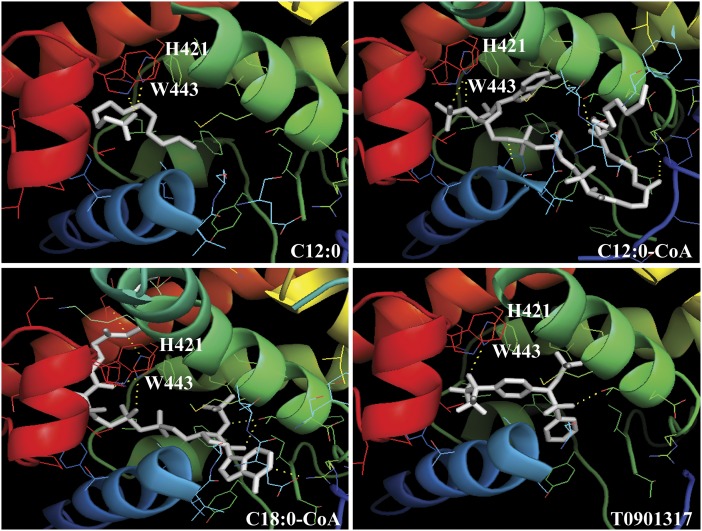
Ribbon diagrams showing the orientation of ligands (white). Lauric acid (C12:0), lauroyl-CoA (C12:0-CoA), stearoyl-CoA (C18:0-CoA), and T-0901317 in the ligand binding pocket of LXRα. Amino acid residues H421 and W443 are shown in stick mode.

Computational methods allow identification of novel ligands for nuclear receptors. Molecular docking was used to investigate the steric and electrostatic complementarity between the LXRα LBD and putative ligands. The availability of LXRα LBD crystal structure allows employment of structure-based virtual screening of various FAs and fatty acyl-CoA (33). The existing structure of the LXRα-RXRβ complex in the presence of T-0901317 (PDB entry 1UHL) was used as a template to screen FAs or fatty acyl-CoA as LXRα putative ligands using AutoDock Vina and SYBYL Tripos. As a first step, LXRα synthetic agonist, T-0901317, was docked as a control to validate the docking parameters. The theoretical docking study of ligands gave results in terms of energy and configurations. As seen in **Fig. 5D**, T-0901317 fits nicely centrally inside the ligand binding pocket with the hydroxyl head group coordinated by hydrogen bonding to H421. This orientation of T-0901317 in the LXRα ligand binding pocket is similar to that proposed by Svensson et al. (33). Docking exercise performed with the FAs and fatty acyl-CoA shows that these ligands similarly orient themselves centrally in the ligand pocket of LXRα. The polar head group of the ligand is situated close to helix 12 and interacts with amino acids H421 and W443 of LXRα in the ligand binding pocket. Whereas lauric acid and lauroyl-CoA ligands completely fit the ligand binding pocket, the hydrophobic tail of stearoyl-CoA is not accommodated in the pocket of LXRα (Fig. 5A–C). The position of docked ligands resembles that of T-0901317 in the LBD of LXRα, as reported in the LXRα-RXRβ heterodimer complex (PDB entry 1UHL) (33). The predicted binding free energies derived by molecular docking listed in **Table 3** gave a similar rank order of binding when compared with the *K_d_* values obtained for the FAs, fatty acyl-CoA, and T-0901317.

**TABLE 3.** The binding free energies of the ligand binding to LXRα

Ligand	AutoDock Vina	SYBYL
T-0901317	−10.8	−2,047
Lauric acid	−5.3	−1,913
Octanoyl-CoA	−9.2	−2,413
Decanoyl-CoA	−8.8	−2,053
Lauroyl-CoA	−7.9	−2,371
Palmitoyl-CoA	−9.1	−2,933
Stearoyl-CoA	−1.6	−2,177

The binding free energies are in kilocalories per mole for the protein-ligand complexes as estimated by AUTODOCK and SYBYL.

### Effect of FAs and fatty acyl-CoA on transactivation of LXRE

**Fig. 6.**
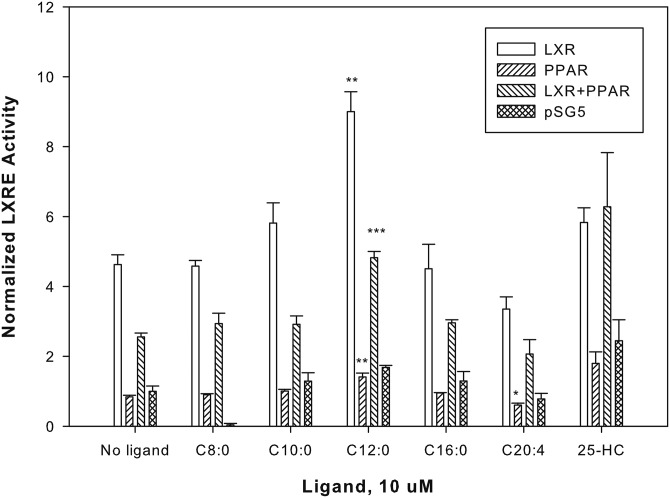
MCFA, lauric acid, or its metabolite, lauroyl-CoA, alter LXRα transactivation. COS-7 cells transfected with pSG5 empty vector, LXRα, PPARα, or both PPARα and LXRα were analyzed for transactivation of the SREBP-1c-LXRE-luciferase reporter construct in the presence of vehicle or 10 μM ligands. The y axis represents values for firefly luciferase activity that have been normalized to *Renilla* luciferase (internal control), where no ligand empty vector (pSG5) sample was arbitrarily set to 1. The bar graph represents the mean values (n ≥ 3) ± SE. *P < 0.05, **P < 0.01, and ***P < 0.0001. Asterisks denote significant differences due to ligand as compared with no-ligand controls. 25-HC, 25-hydroxycholesterol.

A nonfluorescent technique used to confirm the functional significance for lipid binding on the activation of nuclear receptors is the use of transactivation assay. To determine the cellular activity of FAs, a cell-based luciferase reporter assay was used to measure the regulation of downstream transcriptional activity in the presence of FAs (varied in chain length and degree of unsaturation). COS-7 cells were cotransfected with pSG5 empty vector, LXRα alone, PPARα alone, or LXRα with PPARα and analyzed for transactivation of an hSREBP-1c LXRE-luciferase reporter construct in the absence or presence of ligands (**Fig. 6**). Cells were treated with ligands, and transactivation was measured as percent firefly luciferase activity normalized to *Renilla* luciferase (internal control). The fold of activation was calculated against a no ligand (ethanol) control. In cells overexpressing only hLXRα, LXR agonist 25-hydroxycholesterol (positive control) significantly increased transactivation. The addition of the octanoic acid, decanoic acid, and palmitic acid resulted in no significant changes in transactivation activity (Fig. 6), consistent with the weak binding affinity of LXRα for these ligands. Lauric acid, or its metabolite, was the only FA that activated the reporter expression by 2-fold. This result is in agreement with the binding studies that show binding of lauric acid and lauroyl-CoA to LXRα. At 10 uM ligand concentration, arachidonic acid lowered luciferase activity compared with the basal levels, consistent with published data that unsaturated FAs antagonize ligand-dependent activation of the LXR (20–22). The enhanced reporter activity is LXRα-mediated, not PPARα-mediated, because PPARα alone or in the presence of FA shows very little change in luciferase activity. These data suggest that lauric acid, or its metabolite, fulfills the requirement of an LXRα endogenous ligand through which FAs regulate LXRα activity.

## DISCUSSION

In the present work, we demonstrate that medium-chain saturated FAs and fatty acyl-CoA represent high-affinity ligands of LXRα that bind at physiological concentrations. Two separate fluorescence-based assays confirmed that LXRα binding to saturated fatty acyl-CoA is a result of specific binding, rather than a nonspecific aggregation of receptor-ligand complexes. Changes in aromatic amino acid fluorescence, one of the most direct methods to study ligand-induced conformational changes, demonstrated the interactions of LXRα with FAs and fatty acyl-CoA. The decrease in intrinsic fluorescence of LXRα supports a change in environment of the aromatic amino acids upon binding with MCFAs and fatty acyl-CoA. We demonstrated a direct molecular interaction of these ligands with LXRα with well-characterized dissociation constants. The intrinsic quenching assay-measured *K_d_* value of T-0901317 (4 ± 1 nM) is in agreement with those reported in the literature (7 nM) (34). Using the same assay, the potency of FAs, as determined by dissociation constants, showed that binding to LXRα occurs at low nanomolar concentrations. Furthermore, the *K_d_* values are close to the reported concentrations of free FAs present in a cell (35). Thus, binding of FAs and fatty acyl-CoA to LXRα occurs at physiologically relevant concentrations. Disagreement was observed between *K_d_* values determined for palmitoyl-CoA binding to LXRα through intrinsic quenching and fluorescent ligand binding assay. This inconsistency may be explained through earlier findings that fluorescent ligands may have a lower affinity than their nonfluorescent counterparts (36).

We measured the relative binding affinities of various FAs and fatty acyl-CoA with respect to C16:0-CoA binding through in vitro competition LXRα-binding assays. The observed competition between the FAs and existing endogenous or synthetic ligands suggests that these ligands bind at a common site. Established LXRα ligands, T-0901317 and 22 (R) hydroxycholesterol, effectively displaced bound BODIPYC16:0-CoA in the receptor competition assay. MCFA (C10:0) and fatty acyl-CoA (C8:0-CoA) successfully competed with BODIPY C16:0-CoA for binding to LXRα at 100 and 200 nM concentrations, respectively. Long-chain FAs, such as docosahexaenoic acid and phytanic acid, did not displace the bound ligand. This finding implies that long-chain FAs or fatty acyl-CoA may bind poorly or bind to a different binding site on LXRα, hence they do not compete with C16:0-CoA for receptor binding. Review of the literature suggests that FAs, particularly long-chain FAs, prevent binding of oxysterols to LXRα (20, 37). This effect may be mediated through FAs competing with oxysterols for the same binding site or allosterically preventing efficient binding of oxysterols in the LXRα ligand binding pocket. Our data suggests that oxysterols and long-chain FAs, more likely, do not share the same binding site. It remains to be investigated whether FAs induce gene expression similar to LXRα ligands or enhance the interaction of the LXRα with cofactor peptides.

We determined the ability of FAs and fatty acyl-CoA to induce changes in the secondary structure of LXRα. We concluded that subtle structural changes in the α-helix content, β-structure, and turns are induced after the binding of fatty acyl-CoA and FA to LXRα. β-sheet content, as determined by circular dichroism at 190 nm wavelength, was significantly altered by binding of LXRα to MCFAs and fatty acyl-CoA. Although lauric acid and lauroyl-CoA binding quenches the intrinsic fluorescence of LXRα, only lauric acid induces a conformational change in the secondary structure, as determined through the circular dichroic spectra. C8:0-CoA, C10:0, and C10:0-CoA binding not only quenches intrinsic fluorescence, but also induces significant conformational changes in the LXRα structure. This finding implies that ligand-induced exposure of the LXRα aromatic amino acids to the solvent may not accompany large conformational changes in the overall structure. Furthermore, our results showed that weak binding of LXRα to long-chain FAs and long-chain fatty acyl-CoA did not affect the structure of LXRα. Even though our binding assays did not show high affinity binding with long-chain FA or long-chain fatty acyl-CoA, circular dichroic spectra reflected very small conformational changes with C16:1 and C20:5. One possible explanation for this finding could be nonspecific binding of these ligands to various domains of LXRα. This finding is hardly surprising because long-chain FAs are PPAR ligands (38).

The ligand-induced changes in the LXRα circular dichroic spectra, however, did not always correlate with the binding affinities of ligands tested. One possible explanation for the discrepancy may be that circular dichroic spectra provide a global average of all structural changes, and it is entirely possible that changes induced in different domains cancel each other. Alternatively, certain ligands may bind nonspecifically to different regions of LXRα and cause differential changes in the overall structure of the proteins.

The structural basis for the selective preference of LXRα for MCFAs and fatty acyl-CoA derivatives and the proposed role of these molecules as LXRα ligands was confirmed through molecular docking of ligands to the LBD of LXRα. The docking modes demonstrated that the ligand binding pocket of LXRα can easily accommodate the medium-chain fatty acyl-CoA, but not the longer FAs. These theoretical findings are consistent with our binding data, suggesting that MCFAs and medium-chain fatty acyl-CoA can fit nicely in the LXRα ligand binding pocket. On the other hand, long-chain FAs and the acyl chains may be too large to fit in the ligand binding pocket of LXRα (volume of 700 A^0^) (33), inhibiting optimal ligand packing.

Finally, transactivation assays demonstrated that LXRα overexpression alone shows hSREBP-1c promoter activity in luciferase assays, presumably through binding to endogenous RXR. Addition of a FA, particularly the MCFA, lauric acid, caused a statistically significant increase in the luciferase reporter assay using the hSREBP-1c promoter in COS-7 cells. Because the levels of free FAs within cells are generally thought to be low and largely bound to intracellular binding proteins, it is possible that lauroyl-CoA, and not lauric acid, may be the true LXRα ligand. Our binding data agrees very well and is in agreement with this hypothesis. Overexpression of PPARα alone was insufficient to activate the promoter, suggesting that the transactivation activity is LXRα mediated. Co-expression of LXRα and PPARα shows repression of transactivation activity observed with LXRα overexpression alone. Taken together, these data support the idea that saturated MCFAs and fatty acyl-CoA are potential LXRα agonists.

In conclusion, different FAs bind differently to LXRα and have distinct effects depending on the chain length and the extent of unsaturation. Future research may explore the possibility that the effects of medium-chain triglycerides in the treatment of metabolic disorders may be mediated via activation of LXRα.

## Supplementary Material

Supplemental Data
