# Supplementary material for: Fatty acid binding profile of the liver X receptor α
Source: J Lipid Res. 2017 Jan 31;58(2):393–402. doi: 10.1194/jlr.M072447 (PMC5282955; doi:10.1194/jlr.M072447)
Supplement: Supplemental Data [file supp_58_2_393__index.html]

Fatty acid binding profile of the liver X receptor alpha — Fatty acid binding profile of the liver X receptor α — Supplemental Data 

# Fatty acid binding profile of the liver X receptor α

## Supplemental Data

- Supplementary Data (.pdf, 383 KB) - Biophysical profiles of LXR interactions with tested ligands.
