# Supplementary material for: Fatty acid binding profile of the liver X receptor α
Source: J Lipid Res. 2017 Jan 31;58(2):393–402. doi: 10.1194/jlr.M072447 (PMC5282955; doi:10.1194/jlr.M072447)
Supplement: Supplemental Data [file 10.1194_M072447_jlr.M072447-1.pdf]

Supplementary Figures:

Table 1. Affinity of hLXRα for non-fluorescent ligands determined by quenching of hLXRα aromatic amino acid fluorescence (K_d) and ligand efficiencies determined by displacement of hLXRα-bound BODIPY C16-CoA (K_i).

Ligand	Chain length: double bonds (position)	K_d (nM)	K_i (nM)
Palmitoleic acid	C16:1 (n-7)	113±64	N.D.
Palmitoleoyl-CoA	C16:1 (n-7)	46±16	N.D.
Stearic acid	C18:0	25±7	N.D.
Stearoyl-CoA	C18:0	>197	N.D.
Oleic acid	C18:1 (n-9)	45±15	N.D.
Oleoyl-CoA	C18:1 (n-9)	>68	N.D.
Linoleic acid	C18:2(n-6)	21±7	N.D.
Linoleoyl-CoA	C18:2(n-6)	>61	N.D.
Arachidonic acid	C20:4 (n-6)	31±9	N.D.
Arachidonoyl-CoA	C20:4 (n-6)	20±9	N.D.
Docosahexanoic acid	C22:6	>80	N.D.
Docosahexaenoyl-CoA	C22:6	25±3	N.D.
22 (R) Hydoxycholesterol		N.D.	1.2±0.3
25-Hydroxycholesterol		17±4	N.D.

Values represent the mean ± S.E. (n ≥ 3). ND, not determined.

Table 2. *Secondary structures of hLXRα protein in the presence of fatty acids and fatty acyl-CoAs*

Ligand	α-helix regular H(r)%	α-helix distort H(d)%	β-sheet regular S(r)%	β-sheet distort S(d)%	Turns T%	Unordered U%
Ethanol	13.9 ± 0.4	11.9 ± 0.2	13.3 ± 0.3	8.8 ± 0	19.6 ± 0.8	32.4 ± 1.5
C16:1	14.0 ± 1.0	11.3 ± 0.9	17.0 ± 3	10.1 ± 0.9	20.9 ± 0.5*	26.5 ± 2.4
C16:1-CoA	14.3 ± 0.6	11.6 ± 0.6	13.7 ± 1.4	9.3 ± 0.3	20.6 ± 0.6	30.2 ± 1.1
C18:0	16.4 ± 3.2	11.7 ± 0.8	10.1 ± 5.1	9.2 ± 0.4	16.9 ± 3.4	35.4 ± 4.8
C18:0-CoA	10.3 ± 1.3	9.7 ± 0.7	19.0 ± 2.4	10.4 ± 0.5	20.1 ± 1.1	30.4 ± 2.0
C18:1	14.0 ± 1	11.3 ± 0.9	17.0 ± 3	10.1 ± 0.9	20.9 ± 0.5	26.6 ± 2.4
C18:1-CoA	14.0 ± 0.2	10.9 ± 0.8	12.0 ± 6	9.0 ± 0.1	18.9 ± 0.2	35.2 ± 0.6
C18:2	14.1 ± 1.1	11.6 ± 1.2	15.0 ± 2	9.4 ± 1.1	20.9 ± 1.1	28.9 ± 1.3
C18:2-CoA	7.4 ± 0.3	8.5 ± 0.1	28.2 ± 9.8	10.8 ± 0.1	25.4 ± 0.4	19.4 ± 9.7
C20:4	13.2 ± 0.2	10.3 ± 0.1	16.1 ± 2.9	10.1 ± 0.1	21.3 ± 0.5	28.7 ± 0.2
C20:4-CoA	13.9 ± 0.6	12.1 ± 0.3	14.21 ± 0.9	8.9 ± 0.2	20.9 ± 0.3	30.0 ± 0.5
C20:5	13.1±0.1	11.6±0.2	14.8±0.8*	9.1±0.1	20.8±0.1	30.5±0.7
C22:5	15.7±1.9	12.2±0.4	10.1±4	8.3±0.6	19.8±1.5	33.8±4
C22:6	15.6±1.1	12.2±1	14±3	9.5±0.8	21±0.4	27.5±1.8
KH_2PO_4	12.3±0.1	9.8±0.3	16.5±1.8	9.7±0.3	19.2±0.3	32.4±0.7
C20:5-CoA	14.2±0.7	12±0.5	14±0.1	8.8±0.4	21.1±0.5	29.9±0.1
C22:5-CoA	13.4 ± 1.1	11.6 ± 0.5	14.9 ± 1.7	9.2 ± 0.3	20.6 ± 0.6	30.2 ± 1.1
C22:6-CoA	14.3 ± 0.4	12.4 ± 0.2	13.2 ± 0.8	8.5 ± 0.1	20.6 ± 0.5	30.9 ± 0.9

Significant difference between hLXRα with solvent compared to the absence or presence of fatty acids or fatty acyl-CoA dissolved in either ethanol or in KH_2PO_4 were determined by t-test * = $P<0.05$, ** = $P<0.01$, *** = $P<0.001$.

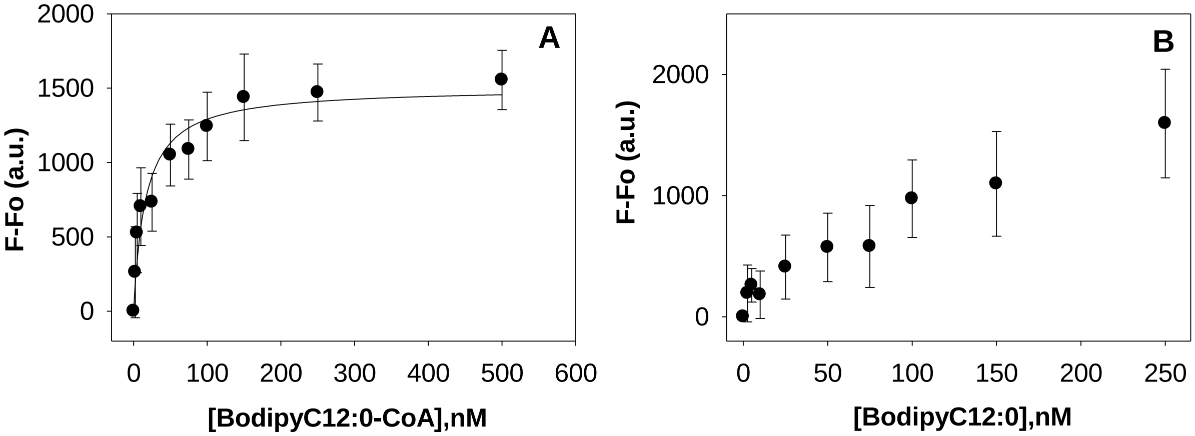

Fig. 1: Forster resonance energy transfer (FRET). Unlabeled LXRα (donor) (excitation wavelength 280 nm) was titrated against increasing concentrations of BODIPY C12:0 or BODIPY C12:0-CoA (acceptor) (emission wavelength 300-540 nm). Changes in the fluorescence intensity at 341 nm wavelength were plotted as a function of ligand conc. to determine apparent dissociation constant (K_d) values.

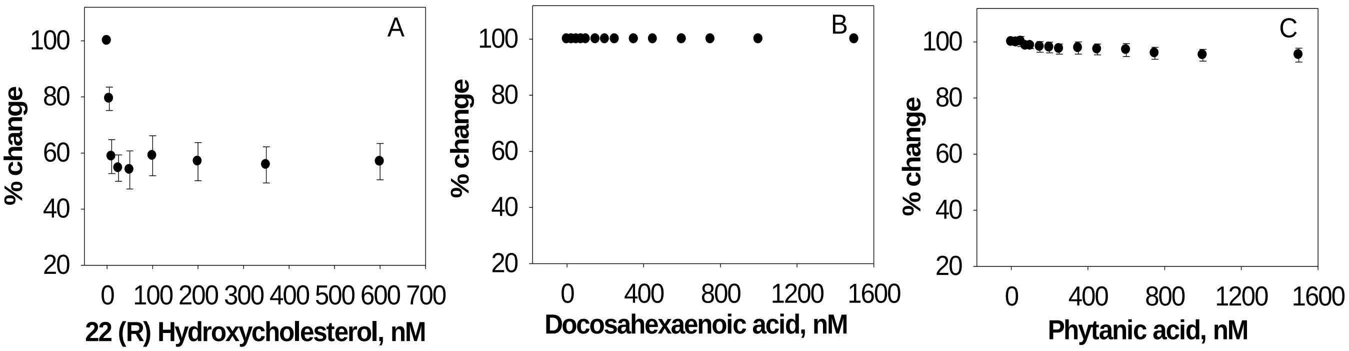

Fig. 2: Displacement assay of BODIPY C16:0-CoA bound LXRα. BODIPY C16:0-CoA bound to LXRα was displaced with LXRα endogenous ligand 22 (R) Hydroxycholesterol, but not with long chain fatty acids docosahexaenoic acid and phytanic acid.

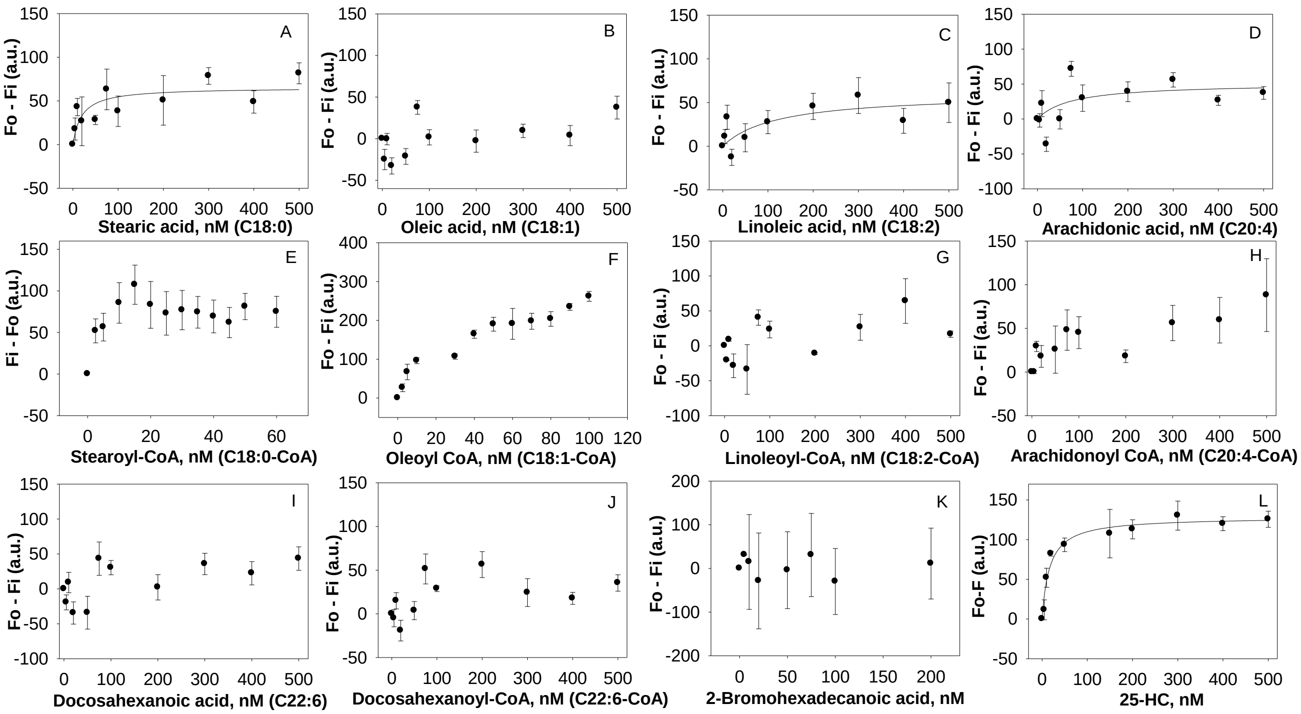

Fig. 3: Direct binding assay based on quenching of LXRα aromatic amino acid fluorescence emission when titrated with the following ligands (A) Stearic acid, (B) Oleic acid, (C) Linoleic acid, (D) Arachidonic acid, (E) Stearoyl-CoA, (F) Oleoyl-CoA), (G) Linoleoyl-CoA, (H) Arachidonoyl-CoA, (I) Docosahexanoic acid, (J) Docosahexanoyl-CoA, (K) 2-Bromohexadecanoic acid, and (L) 25-Hydroxycholesterol

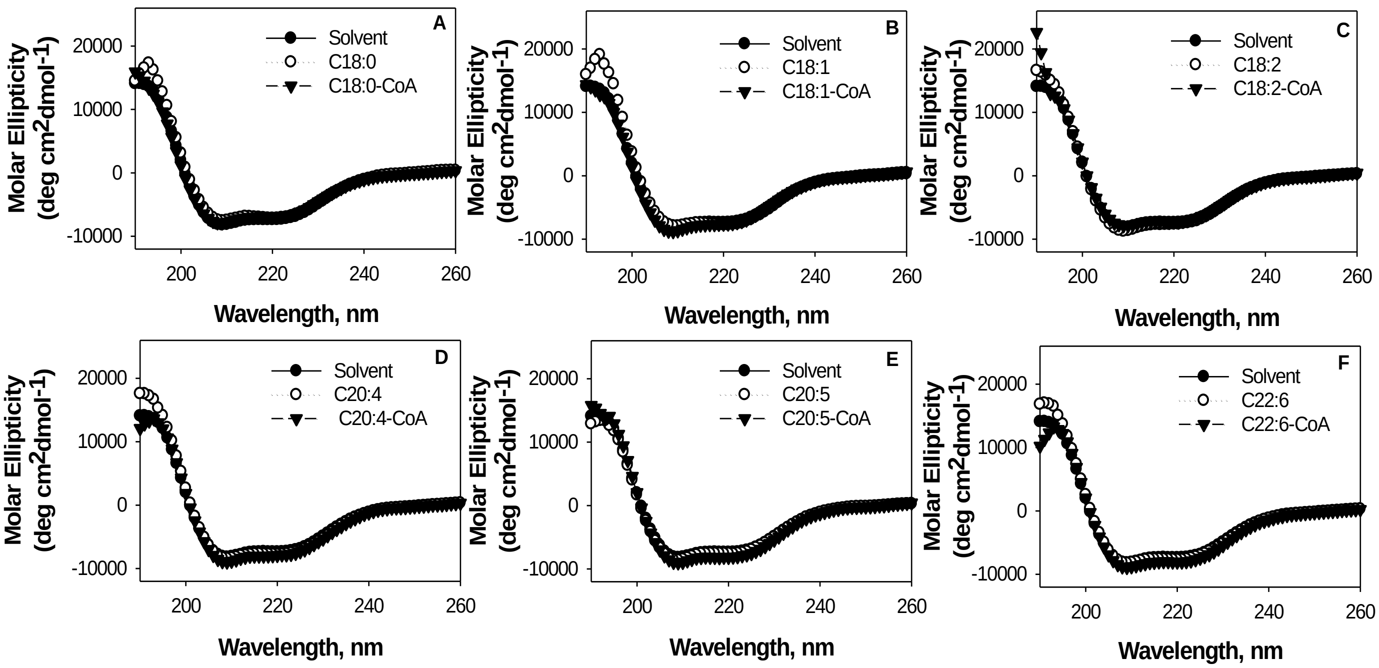

Fig. 4: Far UV circular dichroic spectra of LXRα in the absence (filled circles) and presence of added ligand: (**A**) C18:0 FA (open circles) or C18:0-CoA (filled triangles); (**B**) C18:1 FA (open circles) or C18:1-CoA (filled triangles); (**C**) C18:2 FA (open circles) or C18:2-CoA (filled triangles); (**D**) C20:4 FA (open circles) or C20:4-CoA (filled triangles); (**E**) C20:5 FA (open circles) or C20:5-CoA (filled triangles), and (**F**) C22:6 FA(open circles) or C22:6-CoA (filled triangles). Each spectrum represents an average of 10 scans for a given representative spectrum from at least three replicates.
